# Land tenure, food security, gender and urbanization in Northern Ghana

**DOI:** 10.1016/j.landusepol.2023.106834

**Published:** 2023-09

**Authors:** Eileen Bogweh Nchanji, Takemore Chagomoka, Imogen Bellwood-Howard, Axel Drescher, Nikolaus Schareika, Johannes Schlesinger

**Affiliations:** a Seed Co West and Central Africa, Accra, Ghana; b Institute of Environmental Social Sciences and Geography, University of Freiburg, Freiburg, Germany; c Institute of Social and Cultural Anthropology, Georg-August Universität, Göttingen, Germany; d Institute of Geography, Friedrich Alexander University, Erlangen, Germany; e International Center for Tropical Agriculture, Nairobi, Kenya; f The Institute of Development Studies, UK

**Keywords:** Land tenure, Urban and periurban agriculture, Food and nutritional insecurity, Gender, Northern Ghana

## Abstract

Links between land tenure and food and nutritional insecurity are receiving increased attention. Nevertheless, urban and periurban dwellers face challenges in accessing land to produce food for subsistence and sale. An ethnographic study and food and nutrition insecurity survey were conducted between October 2013 and November 2014 in Tamale, Northern Region of Ghana, to explore the dynamic and recursive links between land access, food access and the ability to maintain resources to meet long-term needs. Results showed that infrastructural development and agriculture compete for land. The shortage of land for agricultural purposes was pronounced in urban areas (20%) than in periurban areas (1.3%) and rural areas (0%). Food insecure households were more likely to name a lack of land than anything else as the primary reason for their inability to grow crops (Fisher’s exact probability = 0.040). Urban and periurban dwellers cope with the constraints posed in the communal tenure system by using strategies such as urban–periurban-rural migrant farming and buffer zone cultivation. The role of women in providing nutritious soups is especially important, and they use various mechanisms to circumvent their lack of access to land and provide food for the household. Political, economic and cultural elements thus interact to constitute the link between land and food.

## Highlights

• Women play a critical role in securing and maintaining household food security.
• Infrastructural development and agriculture compete for land.
• Farmers are using ingenious methods to access interstitial urban and periurban spaces to continue cultivation.
• The shortage of land for agricultural purposes was pronounced in urban than periurban and rural areas.
• Food-insecure households named the lack of land as the primary reason for their inability to grow crops.

## 1 Introduction

### 1.1 Background

Land is a critical resource to socioeconomic development in the Global South, especially in Sub-Saharan Africa, home to more than 60% of people directly depending on the agricultural sector (Goedde et al., 2019, Feyertag et al., 2021). Coincidentally, millions of people suffering from chronic food insecurity and undernourishment live in sub-Saharan Africa (Shimeles et al., 2018). The high prevalence of hunger in the region is attributed to the underperformance of agriculture, climate change, civil and political instability, and high population growth rate (OECD-FAO, 2016, Shimeles et al., 2018). Reversing the food problems in sub-Saharan Africa does not, however, require shifting investment or policy focus to alternative sectors. Instead, transformations that position agriculture on a growth trajectory are required. Central to the transformation is land use and management which, according to Kamau et al. (2021), is critical for the achievement of sustainable development goals (SDG) of ending poverty (SDG 1), zero hunger (SDG 2), and responsible production and consumption (SDG 12).

Land use is the utilization of land resources by populations for diverse purposes, including social and economic functions. Agriculture is an economic and anthropogenic activity that drives changes in land use across regions, directly affecting livelihood, social, and economic outcomes (Ahmed et al., 2016). Much of the recent changes in land use have occurred in sub-Saharan Africa, particularly resulting from rapid population growth and urbanization. High population growth has caused expansion of cultivated land area and agricultural intensification due to increasing food demand (Aleman et al., 2016), while urbanization causes the conversion of agricultural land to commercial, industrial, and residential infrastructure to accommodate populations from rural areas, as well to allow economic diversification of African economies (Magigi and Drescher, 2010, Schoneveld and German, 2014, Kleemann et al., 2017). These changes are responsible for local and regional climate change, soil degradation, and loss of biodiversity which are also linked to food insecurity and malnutrition. This implies that land use is also directly and indirectly linked with SSG 11 (sustainable cities and communities), SDG 13 (climate action), and SDG 15 (reverse land degradation and halt biodiversity loss) (Kamau et al., 2019). Thus, the relationship between land use and social, economic, and environmental impacts is complex and has varying implications across sub-Saharan Africa.

Land tenure is an important social and economic concept that refers to the way land rights are distributed or bestowed to individuals or groups of people, legally or customarily. Tenure incorporates the rights of individuals to access, use, manage, make profit or loss, transform, and transfer ownership of land and land resources. Consequently, land tenure security is linked to land use and access, driving farmers' livelihood diversification options and food security outcomes (Keovilignavong and Suhardiman, 2020). The conceptual linkage between land tenure and food security is explained in literature as being intertwined and land use and productivity are central to the relationship (Borras et al., 2015, Holden and Ghebru, 2016). Land degradation, for instance, causes a shortage of productive land and reduces agricultural productivity which is a threat to food security (Utuk and Daniel, 2015). Therefore, land tenure defines how land is used, playing a crucial role in the ability of individuals and groups to improve productivity and food security.

However, land tenure is important when it secures and promotes inclusive socio-economic development for the achievement of SDG 5 (gender equality). In sub-Saharan Africa, tenure security for women is held back by social and cultural norms which often undermine their livelihoods. Customary tenure is common in Africa, where land is inherited or held by a clan, making land ownership unequal for women (Nadasen, 2012). Married women are often not recognized as part of the lineage, thereby limiting their access to and use of land (Yaro, 2010). Secure land ownership among women also depends on sociodemographic and economic characteristics, including marital status (married, divorced, or widowed), women’s position in households and communities, age, sex, marriage types, education, economic status, and social capital and networks (Nnoko-Mewanu, 2016, Doss and Meinzen-Dick, 2020). Even though policy and legal frameworks have been adopted to address inequalities in land tenure in sub-Saharan Africa, they are insensitive and treat women as a homogenous group with similar challenges (Chigbu et al., 2019). This is not always true given the different circumstances and experiences of women in their pursuit to access land for agricultural purposes.

### 1.2 Setting the context

West Africa is a climate hotspot in sub-Saharan Africa, with changes in land use and technology adoption identified as crucial to averting adverse effects of climate and weather variability on agriculture. Although climate change is cited as an important challenge to food security in West Africa (Ahmed et al., 2016), demographic changes have historically contributed to concerns about food production and security. High urbanization in West Africa is complicating the region's potential to meet the current and future food demand of the growing population. Ghana is a relevant example of countries in the sub-region that has registered rapid population growth in the post-independence era (Kleemann et al., 2017). Much of the urbanization in Ghana is happening in the coastal and inland cities of Accra, Kumasi, and Tamale. In 2020, Ghana had the largest share (57%) of the urban population than any other populous country in the sub-region (World Bank, 2022). The high population in urban and rural areas has caused extreme pressure on land, with significant changes in land use. This presents a major concern in Northern Ghana where agriculture is a dominant activity and is affected by erratic rainfall and long dry periods, causing extreme poverty and chronic food insecurity and malnutrition (Kleemann et al., 2017, Nkegbe et al., 2017, WFP, 2021). The threat of food insecurity is compounded by increasing competition for land for agricultural and commercial purposes (Schoneveld and German, 2014).

The distinction in regional economic development in Ghana between the Northern and Southern parts is attributed to disparities in national investment and differences in natural resource endowment. The historical background of land planning and tenure could explain the differences in levels of economic development in the two regions. Three broad categories of land tenure or rather ownership exist in Ghana: customary, state, and shared ownership. Customary ownership constitutes about 78% of the total land in the country (Schoneveld and German, 2014). Ghanian land legislation recognizes customary ownership and forbids the sale, but only allows temporary alienation through leasing and can be inherited by individuals, groups, sub-groups, or allocation by the chief. Few usufruct titles are held as individual landholdings, meaning that land ownership and access are via groups or sub-groups (Schoneveld and German, 2014).

The chiefs are powerful custodians of land traditions in the north than in the south. Chiefs give land to titleholders, allocate vacant land to users, and arbitrate land disputes. The growing population pressure in the north has therefore created conflicts with chiefs (Schoneveld and German, 2014). In contrast, local institutions in southern Ghana are more experienced in land matters, making land allotment less controversial. In cities, land planning is largely under statutory tenure and, therefore, land management and use are better controlled than in rural areas. However, peri-urban areas are marked by transitions from customary tenure to statutory tenure and the demarcation of the applicable tenure is not clearly defined, leading to tenure issues. The majority of land owned in peri-urban areas, especially land-titled under customary tenure, remains undocumented because the statutory system is considered a colonial heritage (Schoneveld and German, 2014). The confusions about the applicable laws and arising conflicts and contestations have implications on land use and the ability of agriculture and food systems to meet the increasing urban demand for food in Ghana.

Like elsewhere in sub-Saharan Africa, there exist unequal land tenure rights in Ghana despite recent land reforms. Sociocultural norms are deep-rooted and continue framing land access rights for individuals and groups. Land tenure is shaped at several levels, including resource allocation systems, social, institutional, and governance (Britwum et al., 2014). The customary entitlement to land arises out of the multiplicity of social relations which strengthens land access and use for others while weakening claims over land for other groups. Social norms define gender roles and power relations which determine the way socially valued resources are accessed and controlled (Britwum et al., 2014). The social norms in Ghana also run deep in institutional and governance structures for land allocation. The end-product is a land tenure that is insensitive to the experiences of heterogeneous groups, especially women, hindering their access and control over land. Women’s access to land also varies in Ghana, with women’s land rights being less autonomous in the North than in the South. Women in the north own smaller and less productive land parcels, and their land use is limited to growing a narrow range of crops, especially vegetables. Thus, the weak and skewed land rights in Northern Ghana create inequalities that stifle food production and food availability, and accessibility (Nara et al., 2020).

Understanding the land tenure-land/use-food security nexus is, therefore, a critical step in informing policy and legal reforms that would create and reinforce the land rights of individuals and groups. Establishing the relationship between land tenure, land use, and food security will expose the complexities of existing tenure systems and the varying realities of men’s and women’s access to land and food security outcomes in Ghana. Nonetheless, the lived experiences of women are not homogenous for they are affected by varying contexts and individual and group characteristics, which are inadequately covered in the empirical literature. This study investigates how gender and land use varies in Ghana and the implication of land tenure and gender on food security. The study hypothesizes that the complex land tenure systems present challenges to urban and peri-urban women farmers in securing long-term access to land and sustaining their agricultural activities. In testing this hypothesis, the study also posits that traditional gender roles and social norms in Tamale impact women's access to land and resources, contributing to household food and nutrition insecurity. Adaptive land-use strategies can help urban and peri-urban farmers overcome land scarcity challenges created by gender insensitive tenure systems, but have implications on soil fertility, land use, and resource management.

## 2 Literature review and theoretical framework

### 2.1 Literature review

The United Nations ratified SDGs in 2015 with a call to people, nations, and organizations to focus on fostering shared and inclusive prosperity for the realization of the seventeen interlinked global goals. The UN progress report years later acknowledged that it would be challenging for the all the SGDs to be achieved by 2030 unless a holistic approach that clarifies the interrelationships between SGDs and considers the complexities involved are actioned (United Nation, 2018). However, it is inconceivable that 17 goals can be achieved simultaneously just like it is nonsensical to focus on delivering the goals separately (Fu et al., 2019). Studies have recognized that the goals are indivisible and used different approaches, including the nexus approach, to explore interactions and causal relationships between SGDs (Gao and Bryan, 2017). Consequently, reviewing literature that accounts for synergies between gender, land tenure, land use, and food security is critical in understanding how causal relationships among the phenomena can influence the achievement of development goals.

Land is a scarce productive resource that can be managed to meet the demand for food and other products. However, as a fixed resource, land is constrained by competition, pressure, and path dependencies that may enhance or create trade-offs in the achievement of SDGs (Obersteiner et al., 2016). Policy options that center on the management of land-based resources have an implication on interactions among multiple SDGs such as gender equality, zero hunger, no poverty, climate action, and sustainable production. Studies have acknowledged interactions among these goals and indicated that policy-driven land-use systems can undo inherent constraints to the achievement of the goals and solve sustainable management concerns (Kamau et al., 2021). Women in sub-Saharan Africa are mostly involved in agricultural activities such as cultivation, planting, weeding, and harvesting crops (Ben-Ari, 2014), making them important in land-use changes. Accordingly, Fonjong et al. (2013) observed that women play a crucial role in the execution of land-related decisions and should be at the heart of farming, conservation, and land management and policy matters.

Literature has provided mixed results with respect to gender and land use. Nigussie et al. (2017) reported that female-headed households apply less capital-intensive land-use practices such as manure compared to men who tend to use capital-intensive practices like the application of inorganic fertilizer and the use of irrigation. The results contrast earlier findings by Pender and Gebremedhin (2008) who reported that female-headed households applied less manure/compost and contour farming compared to male-headed households. Explainers of the gender difference in land use management practices are cited as differences in physical and human capital endowments (Teklewold et al., 2013).

Besides, land tenure has been cited as an important factor affecting land use, but the evidence remains mixed (Asaaga et al., 2020). For instance, several studies in Ghana and elsewhere in sub-Saharan Africa have found that the intensity of investment in land-use practices varies depending on tenancy agreements (Abdulai et al., 2011, Abdulai and Goetz, 2014). Even so, Asaaga et al. (2020) note that land tenure arrangements in Ghana do not solely influence land-use practices but rather other extra-tenurial context-specific factors such as ethnicity and gender. This suggests socially differentiated land users. For instance, the implementation of sustainable land-use practices in Ejura Sekyedumase and Bongo districts of Ghana was lower among vulnerable and marginalized groups such as women and immigrants due to insecure land tenure (Antwi-Agyei et al., 2015). In most communities in the Global South, land-use decisions are mostly biased against women who struggle due to limited access and insecure control over land because of local customs and culture (Fonjong et al., 2013, Meinzen-Dick, 2019).

The effects of land-use change and tenure security are socially differentiated resulting in differences in productivity and other downstream impacts such as food security. The conversion of agricultural land for commercial purposes has been rapid over the five decades. Agricultural land-use changes have reduced farmland under food production, resulting in lower yield and food insecurity, especially in communities with weak land tenure rights (Appiah et al., 2019, Bonye et al., 2021). Nara et al. (2021) found that strengthening land rights, especially traditional land rights and tenure, enhanced productive land use, thereby leading to increased food production and food security in Northwest Ghana.

The food-security and gender nexus in Ghana is articulated by Fonjong, Gyapong, 2021) who argued that achieving SDG 2 requires gender-inclusive land tenure. Dispossession caused by land-use changes affects women more than men due to women’s close relationship with household food provisioning through farming (Fonjong, Gyapong, 2021; Dzanku et al., 2021; Wood et al., 2021). The intersection between gender and food security in Ghana was also reviewed by Wood et al. (2021) who showed that women have been historically disenfranchised by social and institutional organizations which restrict them from accessing labour and capital outside their homes. However, acknowledgement of women’s role in Ghana has led to some policy progress that aims to erase factors that restrict women from contributing to food security. Food security, land use, and gender equality nexus and its significance in the achievement of SGDs 1, 2, 5 and landscape management are closely linked and can be achieved through land reforms that are inclusive and sensitive to local needs (Asiama et al., 2021). Therefore, the need to integrate a gender dimension into achieving zero hunger via sustainable land use through secure land tenure is critical.

### 2.2 Theoretical framework

The intersectionality framework provides an overarching theoretical framework for analyzing gender, land use, land tenure, and food security nexus. The framework posits that social categories intersect or interact at an individual level to influence social and economic outcomes (Akimowicz et al., 2022). The intersectionality framework is founded on the belief that interdependencies among social categories result in the marginalization of some groups of people within communities. The framework allows exploration of social and economic structures of people's lives and how local contexts create hierarchies and bestow power within societies and their implications on access to resources and services, as well as the impact on all aspects of living in marginalized communities.

The successive development of the intersectionality framework has enabled its application across disciplines, including agriculture. In agriculture and social sciences, the theory is applied to identify marginalized groups and the unique challenges they face in their local contexts, and how the challenges are linked to social organizations (Wood et al., 2021). In the current study, the intersectionality approach provides an accurate picture of the social, economic, and institutional challenges women face in Ghana with respect to land rights, security of land tenure, and land use. The challenges are deeply rooted in customary land tenure systems that perpetuate gender inequalities in land access and use. Formal land access rights are affected by customs which weaken the implementation of inclusive policies. Consequently, unequal land right affects women’s investment in sustainable land management practices resulting in a socially differentiated outcome such as food security. For instance, increased competition for land for commercial purposes has changed agricultural land use in the Global South, causing an increase in food insecurity. Therefore, the intersectionality framework helps in framing the gender-land use, land tenure security, and food security in Ghana.

## 3 Material and methods

### 3.1 Method

**Fig. 1:**
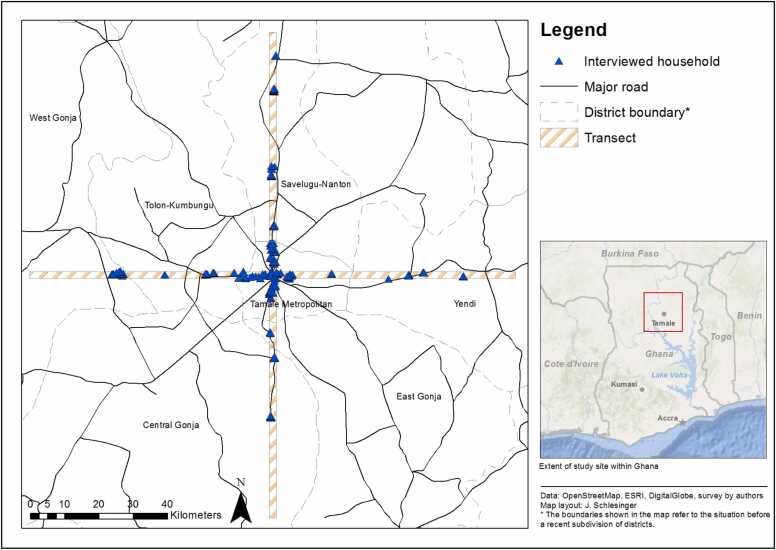
Location map of the study area.

A sequential mixed-method approach was used in this study. Although it can be challenging to meet the assumptions of both quantitative and qualitative research methods, the mixed methods approach used strengthened and maximised the advantages of both methodologies. It provided a more meaningful interpretation of farmers' livelihood strategies in the face of urbanisation and changing tenure systems. A survey was carried out between November and December 2013 in and around Tamale (Fig. 1). The survey aimed at understanding the dynamics of food and nutrition insecurity and the role played by urban, periurban and rural agriculture along the urban-rural continuum. This survey involved 240 households randomly selected over seven districts (Fig. 1). Structured questionnaires were used to collect data on crop and livestock production and consumption and the prevalence of household food and nutrition insecurity.

Simultaneously, an ethnographic study was carried out from October 2013 to November 2014 in and around Tamale to understand the resource use politics of urban and periurban vegetable farming. During this ethnographic study, 12 households were randomly selected from 240 households surveyed for food and nutrition insecurity and men and women in each household were interviewed on how they access land, grow food and provide livelihood for their families.

### 3.2 Sampling

The food and nutrition insecurity survey was based on a transect approach. The transect approach has been used in previous studies on vegetable production, the use of natural resources and the analysis of vegetation (Alberti, 2008, Kamga et al., 2017). Transects were laid radially, 70 km long and 2 km wide from the Tamale central market (Fig. 1). The working definitions of urban, periurban and rural areas were based on reviewed literature on West Africa (e.g. Adam, 2001, Drescher and Iaiquinta 2000). In this study urban areas are defined as those that extend up to 10 km from the city center, periurban 10 – 40 km and rural areas 40–70 km. All the houses along the transects were digitised in ArcGIS 10 Geographical Information Systems software using recent imagery. Twenty households were randomly selected per transect using GIS, giving a total of 240 households being selected (20 households’ x 3 areas/zones x 4 transects).

Twelve households were randomly selected from the 240 households who participated in the food and nutrition survey covering urban and periurban Tamale, six from urban and six from periurban areas, to find out how land tenure systems influence their food and nutritional insecurity. 5 men and 7 women were interviewed with one representative from each household chosen as a key informant, resulting in a sample of 12 participants.

A further series of targeted in-depth interviews and informal conversations with key informants was carried out from December 2013 to November 2014, specifically to delve deeper into the link between access to land and food security in urban and periurban Tamale. The current paper uses data from both studies.

### 3.3 Study food and nutrition indicators

The food and nutrition insecurity study used the following households’ food and nutrition indicators:

Household Food Insecurity Access Scale (HFIAS): this is based on responses to 18 questions about behaviours and attitudes related to food insecurity experience over the past four weeks (consisting of 9 occurrence questions and 9 frequency-of-occurrence questions), resulting in households being assigned scores that range from 0 to 27 (Coates et al., 2007). Households were divided into two HFIAS classes, based on the distribution in the sample as recommended by FAO (2011), with a score of ≤ 11 as food secure and a score of > 11 as food insecure (Chagomoka et al., 2018). A higher HFIAS score reflects greater household food insecurity and poorer access to food.

Women’s Dietary Diversity Score (WDDS): this is a proxy for household nutrition (FAO, 2011). Based on food items consumed in the past 24 h, respondents were assigned the number of food groups they consumed, ranging from 0 to 9. An increase in the number of food groups or WDDS is related to increased nutrient adequacy of the diet. Households were classified into three groups based on the distribution in the sample: ≤ 3 food groups as lowest dietary diversity, 4 – 5 food groups as medium dietary diversity and ≥ 6 highest dietary diversity (Chagomoka et al., 2016a).

The data collection tools were tested for validity and reliability before the actual data collection exercises. Content validity was used to ensure data collection tools' validity. Two subject matter experts reviewed the questionnaire for relevance and completeness of the content. Cronbach's alpha was used to test the internal consistency of our survey questionnaire. The Cronbach's alpha value for HFIAS was 0.83 and for WDDS was 0.79. These values were considered good measures of reliability of the scales in measuring food security because they were above the acceptable level of 0.70.

### 3.4 Data management and analysis

Data was entered with Epidata version 9, and exported to Stata 11 software for analysis. The Pearson chi-square test was used to test the association between reasons for not growing crops and the geographical location. Fisher’s exact chi-square test was used where expected cell frequencies were less than 5 and N < 50. We did the Fisher’s chi-square exact test for household nutrition and food insecurity indicators associated with reasons for not growing crops. Interview guides were used in directing discussions with informants. All in-depth interviews and focus group discussions were recorded and transcribed using the F4 transcription tool.

### 3.5 Ethical considerations

In each community, study objectives and purpose were clearly conveyed to community leaders and respondents. Permission was sought before data collection from local leaders and respondents. Respondents had the opportunity to stop participating in the research at any time of their choice during interviews but none opted out during this study.

## 4 Results

### 4.1 Socio-demographic profile of the study sample

The Dagomba (70%) and Gonja (17%) ethnic groups formed the majority of respondents who took part in the food and nutrition survey which informed the sampling population for further in-depth interviews. Women (39%) and men (61%) participated in the food and nutrition survey (Table 1).

**Table 1:** Demographic characteristics of respondents.

Characteristics		Urban % (n = 80)	Periurban % (n = 80)	Rural % (n = 80)	Total % (n = 240)
Gender	Men	53	65	66	61
	Women	48	35	34	39
Age class of respondents	≤ 20 years	3	1	0	1
	21 – 59 years	90	96	100	96
	≥ 60 years	8	3	0	3
Level of education	None	50	75	79	68
	Primary	4	0	6	3
	Secondary	25	10	5	13
	Tertiary	15	5	0	7
	Koranic	6	9	10	8
Household Religion	Muslim (M)	88	88	99	91
	Christian (C)	11	8	0	6
	Mix M + C	1	5	1	3
Ethnic group	Dagomba	80	64	66	70
	Gonja	3	24	25	17
	Fulani	1	4	9	5
	Dagati	4	0	0	1
	Others	13	9	0	7

### 4.2 Association between the reason for not growing crops and geographical location

The results of the food and nutrition survey revealed that 7.1% of respondents were not producing crops because they did not own any land. Unsurprisingly, land shortages were more pronounced in urban and periurban areas than rural areas (Table 2).

**Table 2:** Association between the reason for not growing crops and geographical location.

		Urban % *( n = 80)*	Periurban % *(n = 80)*	Rural % *(n = 80)*	Total % *(n = 240)*
Growing crops	Growing crops	55	93.8	100	82.9
Reasons for not growing crops	No capital	2.5	1.3	0	1.3
	No land	20	1.3	0	7.1
	Not interested in farming	1.3	0	0	0.4
	Sickness	2.5	1.3	0	1.3
	Trading	3.8	0	0	1.3
	Working	15	2.5	0	5.8

Pearson chi-square = 69.53, (d.f) = 18, P < 0.001.

### 4.3 Association between reasons for not growing crops and household nutrition security

Food insecure households were more likely to name a lack of land than anything else as the primary reason for their inability to grow crops (Table 3). Nevertheless, there was no statistically significant association between various reasons for not growing crops and household nutrition security (WDDS) (Table 4).

Hypothesis 1 *The complex land tenure systems present challenges to urban and peri-urban women farmers in securing long-term access to land and sustaining their agricultural activities*.

**Table 3:** Association between reasons for not growing crops and household food insecurity.

		Food insecure HFIAS> 11	Food Secure HFIAS≤ 11	Total
Reasons for not growing crops	No capital	1	1	2
	No land	5	11	16
	Not interested in farming	1	0	1
	Sickness	2	0	2
	Trading	1	2	3
	Working	1	11	12

Fisher’s exact probability = 0.040 (significant).

**Table 4:** Association between reasons for not growing crops and household nutrition security.

		Lowest dietary diversity ≤ 3WDDS	Medium dietary diversity 4 – 5 WDDS	Highest dietary diversity ≥ 6 WDDS	Total
Reasons for not growing crops	No capital	1	0	1	2
	No land	1	12	3	16
	Not interested in farming	0	1	0	1
	Sickness	0	1	1	12
	Trading	1	1	1	3
	Working	3	3	6	12

Fisher’s exact probability= 0.102 (insignificant).

### 4.4 Land tenure systems in Northern Region of Ghana

According to Nchanji (2018), the land tenure system in the study region is communal, with some pockets of public land. These public lands host government and public buildings which help provide for the socio-cultural and economic needs of the people. In this communal system, land belongs to communities and families. The chiefs are custodians of community lands in trust for the people while the lineage heads are in charge of their family land. Chiefs have allodial rights^1^ over the community land and the farmers have usufruct rights over these same lands, which they can pass on from generation to generation legally. In this system the chief who is the custodian of land for the people is expected to use it on behalf of and in trust for the subjects in accordance with customary law and usage. In this case the chief could lease community land and the proceeds are used for the development of the community. Land used for agricultural purposes in urban and periurban areas can be acquired with a gift token of “kola” (kola here varies in form as it can be kola nuts, bread etc.).

When this is done, the “new owner” has “user’s” rights on the land. If the indigene needs land for a residential building, the same rule for acquiring agricultural land applies. But an additional monetary token is expected after which the indigene is given an allocation note from the chief stating that the land now belongs to him/her. After this process, the indigene can apply for a land title, which is a long term lease. Prices for 100 m x 100 m of land in periurban areas ranged from 2500 to 5000 new Ghana Cedi in 2014 (1 Ghana Cedi was approximately 0.25 Euro in October 2014), while in the urban areas land prices ranged from 8000 to 15,000 new Ghana Cedi in 2014. These prices have definitely increased as of 2018. Land given out just for “kola” can also be leased to prospective buyers prepared to give cash as well as “kola”, if they intend to use the land for development. Lands leased with an allocation note are generally used for the construction of houses and not for any farming activity, as observed by the authors. Most land owners highlighted in interviews that land where persons who had invested to get an allocation note were secured for posterity and not to be sold, even in a case of extreme food crisis. They argued that land is a symbol of identity and pride to be inherited and never sold.

Hypothesis 2 *Adaptive land-use strategies help urban and peri-urban farmers overcome land scarcity challenges, but have implications for soil fertility, land use, and resource management*.

### 4.5 Strategies for securing land

This study revealed that most periurban farmers sought ways to secure their agricultural land in the face of uncertainties about long term access within the communal land system. After the harvesting of cereals and tubers the farmer gives about 100 kg of the crop (usually referred to as ‘kola’) to the chief to maintain ties of trust and loyalty and in so doing secure the use of the land for the next season, similar to land buyers who provide “kola” with additional cash when they want to acquire land for residential and commercial purposes. If farmers fail to provide their land owner with some produce or money because they do not have enough produce to feed their household, the land might be taken from them and given to someone else. The chief usually appoints elders whose role is to check that farmers give him a share of their harvest, which he will use for his family’s needs. This mechanism mitigates against food security in these communities.

**Fig. 2:**
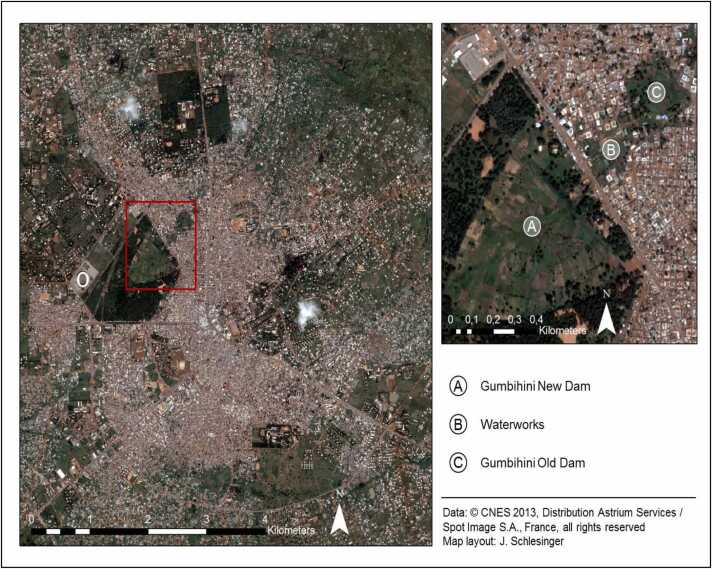
Location map of buffer zone areas in Tamale.

In urban Tamale, there are zones where the Town and Country Planning Department (TCPD) authorities prohibit residential construction following a flood that happened in 1989. The ownership of these lands is contested between different factions of the traditional royal family, the Ghana Water Company (GWC) and the Volta River Authority of the Northern Electricity Distribution Company (VRA/NEDco). The government institutions have won ownership of these lands in the court of law but are not using these lands because they have been designated by the Town and Country Planning Department as disaster zones. The chiefs still maintain that these lands were not sold to the government and are “unofficially” leasing these plots of lands from these zones to any interested buyer. These lands are the Gumbihini old dam, Gumbihini new dam and the former Gumbihini Volta River Authority site (also known as “Waterworks”) (Fig. 2).

The number of farmers who are using these lands for agricultural purposes has continually increased after the disaster as stated by one of the farmers on this site during an interview. Alongside these conventional mechanisms, farmers are working with non-governmental organisations and some government institutions like the TCPD to stake claims to these zones. They are collaborating with various institutions including the NGO - Urban Agriculture Network (Urbanet) to facilitate infrastructural development in some of the sites. Specifically, water pipes have been installed for vegetable irrigation at the Gumbihini old and new dam sites. Interview data from farmers and court officials revealed a colloquial perception that such infrastructural investment in land strengthens one’s claim to it for future use. The main users of these lands are dry season farmers who produce vegetables including cabbage (*Brassica oleracea)*, lettuce *(Lactuca sativa)*, amaranth *(Amaranthus spp),* roselle *(Hibiscus sabdariffa)*, jute mallow *(Corchurus olitorious)* and okra *(Abelmoschus esculentus)*. The buffer zone covers approximately 125,000 m^2^. Besides easing the pressure of land shortages, the use of buffer zones provides a wide range of vegetables to the urban and periurban population.

Farmers have also taken up cultivation on other vacant areas of land outside the buffer zone, for example at Choggu cheferuguni, Ganasco dam, Sangani, and Zagyuri amongst other locations, where vegetables like cabbage, cowpea *(Vigna unguiculata)* lettuce, amaranth, roselle, jute mallow, pepper *(Capsicum annuum)* and tomatoes *(Solanum lycopersicum L)* are grown. These open spaces are found on undeveloped public, private and community lands to which the farmers do not have allodial rights but are squatting, renting, borrowing or have usufruct rights. Some of the farmers have borrowed land from its legal owners and are acting as caretakers to secure it from encroachment.

### 4.6 Land, agriculture and soil fertility

Shortage of land in the urban and periurban areas was frequently due to community land being sold for development, which implies that farmers often produce crops on less than half a hectare of land. Also this has pushed some farmers to crop in and around buildings. This practice has led to depleted and poor soils on farmers’ fields, lowered yields and contributed to household food shortages. In an attempt to overcome this problem, farmers are using several options. They practice mixed cropping with nitrogen fixing legumes such as cowpea to improve the soil’s fertility, and use inorganic fertilisers such as sulphate of ammonia and different blends of compound NPK fertiliser, which, even though relatively expensive, are readily available in the markets. There is also a sizable proportion of farmers using organic manures such as cow dung, chicken manure and sewage as well as compost to improve soil fertility. Apart from boosting the soil farmers also use pesticides in an endeavour to boost production, but due to high levels of illiteracy in the study areas (Table 1), the recommended rates of pesticides are not always applied and some farmers use non-recommended combinations.

Hypothesis 3 *Traditional gender roles and social norms impact access to land and resources, contributing to household food and nutrition insecurity*.

### 4.7 Gender and land ownership

From interviews we confirmed that household heads (‘landlords’) and owners of land were almost always men in the study area. During the main farming season women were usually given a small portion of land on the farm around the edges of their male relatives’ field to produce or cultivate vegetables. This plot of land was often less fertile and considered not “good enough” for cereals or legumes production, which are the main crops. The vegetables cultivated are usually jute mallow, roselle, pepper and okra. This way of growing vegetables by women and sometimes preserving them for use in the dry season is a strategy for improving household food and nutritional security.

Box 1 **Women negotiating land tenure security.**

Nina (name has been changed for ethical reasons), is a widow in a periurban village called Jimle. She lives with her aged mother and children and borrowed land from her brother. Nina complains that this land is infertile. She would like to borrow more productive land from elderly men who have larger surface areas of lands and cannot afford to cultivate them due to high input costs. However, she has been unsuccessful in negotiating access to such lands. She exercises her resource gathering rights by collecting sheanut and dawadawa fruits from communal holdings. She processes these into oil and spice, used for domestic consumption and also as a source of income. Nina also got permission from her brother to fell neem trees found on his land, which she sells as firewood to sustain her family. She considers that non-family members are kinder to women with no land than family members. Nina argues that borrowed land is secure if the borrower maintains a good relationship with the owner. This involves giving some crops, gifts or other basic commodities like salt to the owner after every harvest. Maintaining a good personal relationship is a starting point for negotiating security of tenure for women.

### 4.8 Women, food and nutritional security

Interview results showed that, in order to provide soup for the household while sometimes not having access to enough land, women often work on the farms of their husband or other male kin during harvesting. After harvesting, a certain portion of the crop is given to the female harvesters. In the case of okra, a bowl of okra or more is given to each woman depending on the number of harvesters. In the case of pepper a basin is given to each woman. These vegetables are usually used in the household by the women to prepare soup. In the case of abundant vegetables given after harvesting, women also sell some to get income to buy spices and salt to prepare the soup in their households. In the case of widows, harvesting cereals and legumes are necessary if they are to feed their household as well as sell to supplement household needs. Although women do not own land and sometimes find it difficult to provide the soup, men usually leave some crops during harvesting which women glean and use to provide the soup. After the farm owner harvests, widows and old women can enter any field with their bowls to harvest the left over cereals.

Another interesting element related to women’s responsibility to provide soup lies in the arrangements of access to two economic trees. These trees are the dawadawa *(Parkia biglobosa)* and sheanut trees *(Vitellaria paradoxa)*. For women to access these trees they have to go through men, as these trees are on land owned by men. The fruit of the dawadawa and sheanut trees are consumed by the community as a spice for soup, porridge and as oil respectively. The dawadawa tree is owned by the chief/sub chief in that community, so in most cases permission needs to be sought for its harvesting. Women also collect sheanut fruits to eat and sell the seeds to individuals or shea butter extraction production centres. They also use shea butter for cooking and pepper preservation. These trees therefore provide income generating opportunities to the women who sell the fruits and their by-products. .

Box 2 **Improving value chains.**

In urban Tamale, around Gumani, securing land for agricultural activities is difficult, as Ashaitu (name has been changed for ethical reasons) notes. Her husband has no piece of land and his former land holdings have been sold by the chief to estate developers for residential purposes. Ashaitu is the sole provider of food for her household. She has multiple activities she engages in to feed her family. Ashaitu harvests on the farms of her friends and kin, and is paid with the crop she harvests. She does not have the luxury of choosing the type of crops she can harvest, so she harvests any crop she is called upon to assist. Ashaitu prefers to harvest rice, cereals, groundnuts and vegetables. She consumes all the vegetables either fresh or in dried form and she processes the rice she harvests and sells it to generate more income. Ashaitu says land ownership is important but not sufficient, as you need other technical farm inputs to be able to get a good yield from the farm to feed the family.

### 4.9 Land and migrant farmers

In the study site, we observed a new phenomenon of urban to rural migration. This information came out of discussions with farmers who described their search for agricultural land in rural areas. This move was prompted by the search of land in areas where urbanisation, population pressure and shortage of arable land are not yet perceived to be a problem. They give “kola” in exchange for agricultural land where they farm. After harvesting they bring their harvest back to the periurban and urban areas for consumption and sale.

Urban farmers, due to land shortage, are also moving their production activities to irrigation sites, where they rent plots of land and pay water charges to grow their vegetables and staple crops for home consumption and income generation. There is an influx of urban farmers from Kumbungu and Tamale in Ghana’s Northern region, and even from Bawku in the Upper East region, to irrigation sites such as Bontanga and Golinga, This usually occurs during the dry season, when okra, onion *(Allium cepa)*, green pepper and rice *(Oryza sativa)* are grown to target the early market, including the festive periods of christmas and new year holidays. Onions are produced in large quantities and are sold in Tamale, Kumasi and Accra amongst others destinations.

## 5 Discussion

This study reveals that chiefs’ manipulation of the customary land tenure system in Northern Region of Ghana is one mechanism whereby agricultural land is lost to residential construction. The manipulation of customary land tenure system by chiefs and the hybrodization of land tenure systems are not only a manifestation of inefficiencies (Nchanji et al., 2017, Nchanji and Bellwood-Howard, 2018), but also its broader implication for food security. These occurrences could be attributed to urban planning policies that encourage rezoning of public land to private developers and residential areas, shrinking available land for agricultural activities and leading to food insecurity. The hybrid planning practices in urban and peri-urban overseen chiefs and local authorities propagate tenure insecurity and encourage land speculation and conversion of agricultural land for commercial purposes (Akaateba et al., 2021), thereby undermining the realization of sustainable cities and communities (SDG 11). The exacerbation of tenure insecurity for peri-urban and urban areas also renders residents landless without alternative sources of livelihood and secure food sources. This relates to findings by Afriyie et al. (2020) who found that spatial expansion of Greater Kumasi reduced the availability of arable land for urban and peri-urban agriculture, denying farmers access to land to meet their basic needs worsening their economic conditions.

Comparison to other urban and peri-urban areas in Ghana reveals almost similar across various the country. Although distinct patterns may be influenced by regional-specific factors such as economic conditions, cultural norms and population density are the popular drivers of land use changes in major urban areas in the country. For instance, like in Tamale, the majority of customary lands in Accra and Cape Coast have been leased to private individuals due to rapid urbanization and increased demand for land. The land tenure system in these cities are characterized by improper documentation of land transactions and boundaries, encroachment on public lands, multiple sale of lands, and intractable land disputes (Water Aid, 2009).

However, in Tamale, land users do not engage in land markets and do not benefit financially from economic transactions involving their lands (Ubink, 2007), being limited to giving ‘kola’ to secure their access to it. This non fungibility of land and cash from the users’ point of view contributed towards the study respondents’ noncommercial conceptualisation of land. Similar strategies were observed by Townsend (1995) in India, where households who did not sell their capital assets instead depleted their cash reserves and adjusted their eating habits. Corbett (1988) and Debessa et al. (2022), also revealed that households did not sell capital assets but instead reduced their food consumption or adopted food coping strategies which would not hinder their household income generation in the long term. Chagomoka et al. (2016b) reported diverfy food coping strategies used by households in West African cities.

However, despite the threat to their ability to access land, farmers in Tamale were using ingenious methods to access interstitial urban and periurban spaces and thus continue cultivation. In addition to counteracting land sales by chiefs with ‘kola’, they cultivate on the buffer zones and other unoccupied urban land (Nchanji et al., 2017). The movement of urban farmers to periurban spaces, including irrigation sites, can also be seen as part of this strategy. All these farmers are exploiting loopholes and gaps in existing tenure arrangements to gain access to the crucial resource, land in order to feed their families.

The urban to rural migration we encountered contrasts with the longstanding phenomenon of rural-urban migration by farmers offering labour for wages. This changing strategy has also been noted by Yaro (2010), who describes a case where farmers migrated to Gbanyamni, a periurban town 10 km from Malshegu, Tamale, and had to further move due to land commodification some years later to a rural area in search of land to farm. In Burkina Faso the Groupe de Recherche et d′Action sur le Foncier (2011) discovered that migration patterns towards the rural areas for land were due to poor soils in urban and peri-urban areas, and pastoralists’ increasing cultivation of fodder crops as part of their livelihood strategy.

Furthermore, the findings of the study challenge the long-standing traditional hypothesis that secure tenure incentivizes agricultural investment. Classically, secure tenure is hypothesised to incentivise intensive agricultural investment (Fatton, 1997). According to this perspective, farmers will be more willing to invest for three reasons. They will be keen to enjoy the returns of long term improvement and conservation measures: the ‘assurance effect’ (Braselle et al., 2002). Returns on investment made can easily be recuperated, which is the ‘realisation effect’, and farming productivity increases through improvements in allocative efficiency. Yet Bruce (1988) questioned the direction of causality between tenure and investment, arguing that tenure security may not cause investment to increase but rather investment may stimulate land security. The study found that farmers' investments in soil fertility were found to be more influenced by socio-economic status than by land security, suggesting that the relationship between tenure and investment may be more complex than previously assumed. This observation is reinforced by a study in Ghana showed that tenure security had a very positive impact on investment in the Anloga area, but a less noticeable impact in Wassa-Amenfi and no influence in Ejura (Migot-Adholla et al., 1994).

Besley, 1995a, Besley, 1995b used the same data to assess the sensitivity of the results to the estimation methodology used, and reached the conclusions that better land rights facilitated investment in Wassa but not in Anloga. In Tamale, interviews with the farmers revealed that as much as access to land influences food and nutritional security, the effects of poverty are also significant. Investments in soil fertility of land are more influenced by the farmer’s socio-economic status and are not directly influenced by the security of access to the land (Nchanji et al., 2017). In fact, influences on soil fertility management are diverse and interact with household roles and responsibilities. Organic manures such as faecal sludge are being used by farmers in Tamale to improve their soil fertility and increase yields to be able to feed their families (Gyasi et al., 2014). In Burkina Faso, when women plant legumes to fix nitrogen in the soil, men sometimes took over the improved soil the next year to plant their cereals (Jones-Casey, 2014). Like the majority of West African farmers in the increasingly common situation of land scarcity, those in Tamale implement diverse soil fertility management strategies to maintain high yields.

The majority of crops grown on these urban farmlands are vegetables, which are good sources of micronutrients and help households to generate income (Chagomoka et al., 2014). Urban agricultural activity does contribute to improved household food and nutritional security (Chagomoka et al., 2018). However, the use of these interstitial spaces for agriculture does have its drawbacks for the consuming urban populace. Pesticide misuse and the occasional use of waste water for irrigation (Kamga et al., 2013, Nchanji et al., 2017) mean that intensive urban and periurban farming has possible health and food safety risks, which occur partly as a result of farmers’ intensive use of chemical inputs on their small spaces of land in the urban zone.

The role of women in maintaining household food security is crucial. Our data revealed that women have used different food security strategies to reconcile their lack of access to land (Nchanji and Bellwood-Howard, 2016). Thus, in a situation where land is becoming scarcer in general, these strategies, involving gleaning and cultivating on field edges and small plots, come to the fore. Women’s cultivation of soup vegetables is not exclusive to the Northern Region of Ghana: in Kenya and Burkina Faso women also cultivate crops perceived as less valuable (Jones-Casey, 2014). Mechanisms have also developed that allow women access to natural resources on land owned by men, concomitant with their role as gatherers (Doss et al., 2014). Their use of fruits and seed for food and as a source of income mirrors their use of other natural resources such as firewood and water. Yet they do not gain ownership over any of these resources. Inter-gender, class and status power relations are especially evident in the case of the dawadawa tree, important to women’s livelihood strategies but considered to belong to the traditional authorities (Mahama, 2009). The activities employed by women to guarantee food security, such as harvesting from male kin, relying on economic trees and cultivating borrowed land, are set to continue and even to gain importance in the future (Nchanji and Bellwood-Howard, 2016a, Nchanji and Bellwood-Howard, 2016b) as more of the urban population loses access to agricultural land.

Although the study underpins the intersection of gender, land insecurity, and food insecurity in Tamale and more broadly in Northern Ghana, the findings lso reveals intricate web of socio-economic and cultural dynamics that can influence food security outcomes. This finding may also resonate to other regions in the country and beyond. In addition, the study reveals that land tenure systems in the Tamale, customary or statute, places women in a precarious position which has an implication on types of policies that can be instituted to address the issue. However, despite women facing hurdles in access to and use of land, they play a crucial role in maintaining household food security. Therefore, the study provides a step towards the understanding of policies that can integrate gender-sensitive strategies and recognition of women roles in food security in provision of solutions to their relative lack of land access.

Furthermore, study reveals local farmers’ resilience and adaptability to land tenure issues in Northern Ghana. Despite facing land use obstacles, farmers find innovative strategies to use urban and periurban spaces for food production. However, the strategies pursued by farmers are a further revelation of larger structural issues related to land commodification, urbanisation, and the changing dynamics of land tenure systems in Northern regions and the entire country to some extent. This has a crucial implication on urban and periurban agriculture policies and land reform, as well as food security. Therefore, although the study is contextually grounded in Nothern Ghana, the insights it provides are valuable in understanding gender, land, and food security nexus in Ghana and similar contexts across the world, especially with respect to dialogues on sustainable cities.

## 6 Conclusion

The study concludes that the communal nature of the land system in Northern Region of Ghana interacts with agricultural and food provisioning activities and consequently affects household food and nutritional security. Growing crops entails not just access to land but also access to other bundles of power associated with financial institutions, inputs and health. Due to the complexity of land access mechanisms in urban and periurban areas, and the commodification of urban land, farmers are adapting various strategies to provide food for their households. These strategies are embedded in social relations and interactions with family and external actors to gain access to buffer zones, irrigation sites, periurban and rural sites.

In the midst of this complex interaction between land tenure and food and nutritional security are women. They do not usually own land, but are expected to provide soup for the household. To cope with this dilemma, they are found working as harvesters so as to have crops like okra and pepper to provide soup for their household. Simultaneously, they seek permission from men to harvest from trees of economic importance such as the dawadawa and sheanut. These food provisioning strategies of the landless may continue in a situation of increasingly difficult access to land and have implications at landscape scale, for example for the conservation of trees.

Land tenure and food and nutritional insecurity are thus embedded in the socio-economic and political environment of urban and periurban farming households. Therefore, understanding the urban and periurban farmers’ context can help to grasp the recursive links between access to land, access to food and the ability to maintain sufficient resources to meet long term needs. Thus, from a policy perspective, understanding and ability to manipulate the flexibility of the communal land system through encouragement of integration of vegetable cultivation and other urban and periurban agricultural activities into the socio-political milieu will go a long way towards improving household food and nutrition security. At the same time, new forms of institutional organisation, particularly in irrigation projects, are opening up alternative modes of access to land for women. These, and other government schemes, should be encouraged to enhance women’s access to resources needed to boost household income and food security.

The findings that customary land tenure system and land commodification have implications on secure tenure for agricultural investments, food security, and inclusive cities and sustainable development should be used to inform both national and local policy decisions. Local and national policies decisions could range from stricter regulations on land conveyancing and adjudication the development of more equitable land tenure systems that consider the needs of local farmers, especially women and immigrants. The findings also provides an entry point for policy that balances urbanization and preservation of agricultural lands for food security and sustainability of urban livelihoods.

Furthermore, the study findings highlight the critical role played by women in securing and maintaining household food security. Understanding of strategies used by women to navigate land access and use constraints reveal need for developing gender-sensitive agricultural interventions. Such interventions could encompass promoting and securing women’s access to land. The program could also entail creating opportunities for women in urban and peri-urban agriculture to engage more in profitable and sustainable farming practices by providing training and resources. Such programs could also form entry points for addressing urban farming challenges, such as potential health risks associated with use of waste water for irrigation and misuse of pesticides. Training and educational programs in sustainable farming practice could ensure adherence to health and safety standards in urban farming. Considering that the urban farming households are often poor and marginalized, implementation of sustainable social safety nets that consider the complexities in accessing and using land and gender issues is recommended. For instance, agricultural support programs that increase access to agricultural training, resources, and credit will not only ensure improve women’s ability to make efficient use of scarce land but also critical in addressing food security in a sustainable way.

The study also acknowledges and provides a firm foundation for future researchers. For instance, future research should explore the socio-economic factors influencing investments in soil fertility and use of sustainable farming practices in urban agriculture, with consideration of both ingenious and modern farming methods. The limitation of the study is its narrow focus on the northern region of Ghana, and therefore may not fully reflect land use and food security dynamics across the entire country due to uniqueness of land tenure systems in each region. However, the issues and trends uncovered by this study are relevant to other countries in West Africa, sub-Saharan Africa, and other developing regions of the world that are experiencing similar dynamics of urbanization, changing land use patterns, and food security challenges. Therefore, the study provides global lessons that can be used by developing countries in responding to these issues. Another limitation relates to limited focus on effect of gender dynamics surrounding land use and agriculture and the effect of specific cultural practices on what was observed by study. Thus, further research would be critical in feeling these gaps.

## CRediT authorship contribution statement

**Schareika Nikolaus:** Conceptualization, Project administration, Supervision. **Schlesinger Johannes:** Project administration, Supervision. **Chagomoka Takemore:** Conceptualization, Formal analysis, Methodology, Writing – review & editing. **Nchanji Eileen Bogweh:** Conceptualization, Formal analysis, Methodology, Supervision, Writing – original draft, Writing – review & editing. **Drescher Axel:** Project administration, Supervision. **Bellwood-Howard Imogen:** Formal analysis, Methodology, Writing – review & editing.

## Declaration of Competing Interest

The authors declare that there are no potential competing interests.

## Data Availability

Data will be made available on request.

## Footnotes

1 Free, not subject to the rights of any lord or superior, owned without obligation of vassalage or fealty
